# A Service-Caching Strategy Assisted by Double DQN in LEO Satellite Networks

**DOI:** 10.3390/s24113370

**Published:** 2024-05-24

**Authors:** Yuchen Luan, Fukun Sun, Jiaen Zhou

**Affiliations:** 1 Aerospace Information Research Institute, Chinese Academy of Sciences, Beijing 100045, China; luanyc@aircas.ac.cn; 2 School of Information and Communication Engineering, Beijing University of Posts and Telecommunications, Beijing 100876, China

**Keywords:** Satellite fog computing, Double DQN, caching hit

## Abstract

Satellite fog computing (SFC) achieves computation, caching, and other functionalities through collaboration among fog nodes. Satellites can provide real-time and reliable satellite-to-ground fusion services by pre-caching content that users may request in advance. However, due to the high-speed mobility of satellites, the complexity of user-access conditions poses a new challenge in selecting optimal caching locations and improving caching efficiency. Motivated by this, in this paper, we propose a real-time caching scheme based on a Double Deep Q-Network (Double DQN). The overarching objective is to enhance the cache hit rate. The simulation results demonstrate that the algorithm proposed in this paper improves the data hit rate by approximately 13% compared to methods without reinforcement learning assistance.

## 1. Introduction

### 1.1. Background and Motivations

With the emergence and widespread adoption of 5G systems, the limitations inherent to terrestrial networks, particularly in terms of the coverage area and construction costs, have become increasingly pronounced. This has spurred a quest for alternative solutions to mitigate these drawbacks. Concurrently, as communication technology continues to evolve and our understanding of space deepens, there has been a burgeoning interest in exploring the potential of low-Earth-orbit (LEO) satellite internet as a viable alternative.

LEO satellite networks offer several distinct advantages over traditional terrestrial networks. Firstly, they provide extensive coverage, spanning vast geographic areas that may be challenging or cost-prohibitive for terrestrial infrastructure to reach. Secondly, the deployment costs associated with LEO satellites are comparatively lower than those of ground-based networks, making them an attractive option for expanding connectivity to underserved or remote regions. Additionally, LEO satellites boast a significant capacity, enabling them to support a large volume of data traffic with minimal latency. Furthermore, the integration of LEO satellite networks with other high-altitude platforms, such as UAV networks, presents opportunities to enhance the delivery of services to ground users. By leveraging the flexibility and mobility of UAVs, satellite internet providers can offer more convenient and tailored services, thereby improving the overall user experience [1].

In the context of future communication technologies, such as 6G, satellite internet emerges as a key breakthrough direction. Its inherent advantages make it well-suited for various applications, including civilian and emergency communication, Internet-of-Things (IoT) connectivity, military operations, and beyond [2,3,4]. As such, the potential for the further development and widespread application of satellite internet is vast and promising [5]. Amidst the construction and deployment of LEO constellations such as Starlink, the conventional LEO satellite service model, which relies on transparent forwarding, is no longer suited to meet the rapidly evolving demands of businesses. Various applications now require lower latency and smoother content delivery, necessitating more responsive and efficient services at the user end. Advancements in storage technology and computing chips have empowered LEO satellites to offer computing and caching services for content. However, the exponential surge in demand for low-latency mobile applications and multimedia services poses a significant challenge. According to Cisco’s Visual Networking Index (VNI) report [6,7], IP video traffic is expected to double by 2022, constituting 82% of the total IP traffic. In response to the escalating traffic demands, LEO satellite caching has garnered attention as an innovative solution. By pre-caching popular content on LEO satellites, which can function as fog nodes, timely and dependable content services can be delivered to edge users [8,9,10,11]. In satellite networks, numerous challenges are encountered, such as the time-varying topology of the satellite network. The neighboring satellites of a satellite vary at different times. Satellites move at high speeds, resulting in increased switching frequencies and lower cache hit probabilities. In the model proposed in the paper, content requested by users is considered a cache hit for both the satellite and its neighboring satellites, which significantly differs from ground-based networks.

The dynamic movement of LEO satellites results in continuous changes in the coverage area of satellites and the connected edge user terminals, necessitating frequent data replacement in the cache [12,13,14,15]. However, the cache space of each satellite is limited, and redundant data may accumulate if identical data segments are repeatedly cached across the satellite constellation, leading to suboptimal cache space utilization. Therefore, exploring collaborative cooperation among satellites becomes imperative [16,17,18]. Additionally, for large-scale LEO constellations, processing massive data caching incurs high computational complexity and costs [19]. To tackle these challenges, the development of a rational LEO satellite data-caching scheme is essential. This scheme should focus on determining cache placement locations and enhancing cache efficiency [20,21,22].

### 1.2. Related Works

Currently, scholars have conducted in-depth research on caching strategies for low-Earth-orbit (LEO) satellites. Some scholars aim to reduce the bandwidth resource consumption when satellites transmit cached content, balancing network load distribution as an optimization objective. The authors in [23] propose a dual-layer caching model based on content delivery for satellite–ground communication. This model deploys one layer of cache at ground stations and another layer on satellites, reducing satellite bandwidth consumption through the joint optimization of the dual-layer cache. The literature [24] presents a caching deployment method for LEO satellite networks based on named data networking (NDN), allocating loads in the network and maximizing the effectiveness of internal caching. This method considers the placement of cached data based on the topology of the satellite constellation. The results show that, by using only a small number of caching nodes, the length of the cache path can be reduced to one-third, aiding in load distribution within the network.

Furthermore, some scholars aim to reduce resource consumption in the network by increasing cache hit rates. Bommaraveni et al. employ active learning methods to understand the popularity of content, allowing the system to balance between caching new content and current content [25]. Xu et al. propose a replacement algorithm called ALIRS to improve scalability, maintaining cache hit rates while reducing service latency [26]. Chen et al. formulated a cache placement problem based on obtained request probabilities, aiming to maximize cache hit rates under storage capacity constraints [27]. They developed a dynamic programming algorithm to obtain the optimal caching strategy.

To enhance satellite service quality for users to obtain a better experience, the authors in [28] propose a quality of experience (QoE)-based optimization scheme for video stream caching placement. This scheme considers the required video stream rates and social relationships among users to optimize cache placement. The results show that the proposed caching method significantly outperforms traditional methods in terms of QoE. The authors in [29] study joint caching placement and content delivery in satellite–ground integrated cloud wireless access networks. The literature minimizes long-term power consumption through optimizing cache placement, access point (AP) clustering, and multicast beamforming. In [12], the authors propose a content layout optimization-based caching algorithm for LEO satellite constellation networks. They designed an optimized caching policy to cache popular content preferred by users on satellites. This scheme enhances service quality in various scenarios and achieves more efficient content distribution within the satellite network.

However, the above-mentioned solutions mainly focus on the optimal caching placement at specific time points, neglecting the cooperation between satellites and resulting in the underutilization of cache space. Therefore, the authors in [30] propose a cooperative content-sharing method between satellites to maximize the utilization of the limited storage space on individual satellites. By increasing the connectivity status between satellites and ground stations to ensure coordinated content transmission, they effectively reduce the average service latency and address challenges using cooperative caching between multiple satellites and base stations. In [31], to jointly consider meeting user resources, the authors model the data-caching problem in satellite–ground networks as a joint optimization problem involving caching, resource allocation, and computational resources, and they solve it using deep Q learning algorithms.

### 1.3. Contribution and Organization

In this work, we employ the Double DQN algorithm to predict service content for LEO satellites. The proposed method aims to improve the cache hit rate of LEO satellites. Moreover, as the cache hit rate increases, the frequency of content requests within the network decreases, thereby reducing the likelihood of communication congestion in the entire network. Additionally, it can decrease the computational resources consumed via the system for content re-encoding and decoding. Within this framework, the main contributions of this paper are as follows:

- We propose a three-layer architecture integrating cloud, fog, and edge computing. The fog layer can provide services to edge users through its own content or content from neighboring nodes while also accessing content from the cloud, thereby achieving synergistic effects among cloud, fog, and edge computing.
- We utilize a reinforcement learning-based approach to train Double DQN agents and conduct simulation experiments based on synthetic data, providing an optimal solution for satellite caching schemes.
- We established a simulation experiment verification scheme and compare it with multiple methods to validate the effectiveness of the proposed approach in satellite edge caching strategies.

The rest of this paper is organized as follows: in Section 2, we present the network model of the cloud–fog–edge three-layer architecture and introduce the caching mode. Section 3 introduces the Double DQN method adopted in this study. Section 4 describes conducted simulation experiments and analyzes the results based on the proposed architecture and method. In Section 5, we summarize the entire work and provide future research prospects.

## 2. System Model

### 2.1. Network Model

**Figure 1:**
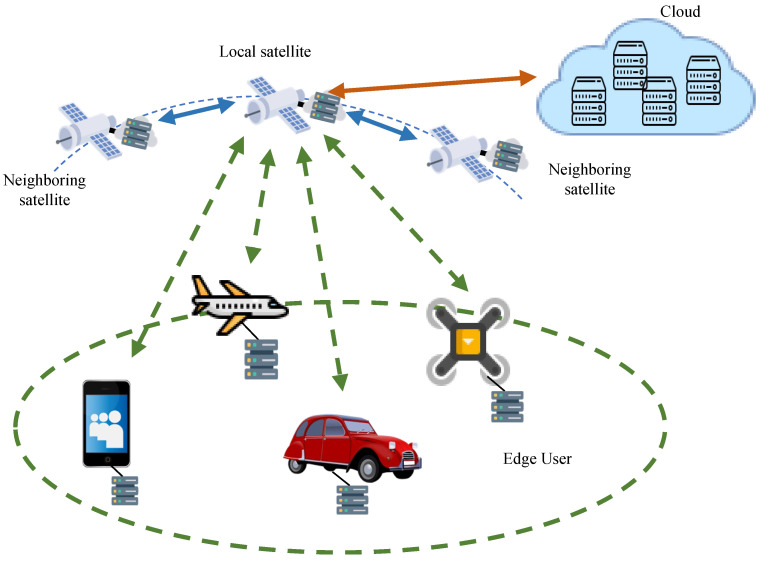
Network model.

As depicted in Figure 1, our work considers a network architecture composed of three tiers: cloud, fog, and edge. The cloud layer comprises service centers deployed on the ground or at medium and high orbits, containing all content and services. The middle layer consists of LEO satellites, which store a portion of content and services and connect to both the cloud service center and edge users through wireless networks. Edge users, such as vehicles, aircraft, and other terminal devices, have demands for content and services. Due to the fast movement of satellites, edge users are assumed to be stationary in the network model, while fog satellite nodes are in a state of high-speed motion. Each edge user locally stores historical data and content preference records. Since fog satellite nodes only cache a portion of content and services, when the cache of a fog satellite fails to match the demands of edge users, it needs to retrieve the corresponding content from the cloud server. Specifically, the following aspects are involved:

1. Fog satellite nodes: Fog satellite nodes directly connect with edge users and provide network and content services. When the requested content is available at a fog satellite node, it interacts directly with the edge user in the form of content or services. If the requested content is not pre-cached, the fog node requests it from adjacent nodes or the cloud service center and caches the relevant content.
2. Adjacent satellites: If adjacent satellites cache the requested content, they collaborate to provide content or services to the satellite directly connected to the edge user and then continue to serve the edge user.
3. Cloud service center: The cloud service center is connected to the backbone internet. We assume it contains all content and services. Thus, when fog nodes are unable to provide content services, they initiate content retrieval requests to the cloud center, cache the content locally, and continue to provide services.

### 2.2. Mobility Model

Due to the complexity of satellite networks, we adopted the BPP model as the representative for satellite networks in this study. The BPP model has been proven by Wang [32] to be an effective model for satellite networks.

**Proposition** **1.** *For a point in homogeneous BPP, the azimuth angle is uniformly distributed between *0* and 2π, i.e., $\phi_{BPP}$∼U[0,2π], and the cumulative distribution function (CDF) of each point’s polar angle (of the spherical coordinate), $\theta_{BPP}$, follows*

(1) $$F_{\theta_{BPP}}\left(\theta \right)=\frac{1-\cos\theta }{2},0\leq \theta_{BPP}\leq \pi ,$$

*and $\theta_{BPP}$ can be generated by,*

(2) $$\theta_{BPP}=\arccos\left(1-2U\left[0,1\right]\right),0\leq \theta_{BPP}\leq \pi .$$

Note that the BPP given in subsequent parts of this paper means the homogeneous BPP unless otherwise stated. The distribution of user terminals is modeled randomly.

Considering the relativity of motion, we assumed that the satellite topology remains unchanged, assuming that ground terminals have a random initial direction and speed of movement for simulation. When a ground user moves into the coverage area of a satellite, they initiate a content request to the satellite. To facilitate simulation, we set up a scheme for each time slot that calculates the positions of ground users before the start of each time slot, and within each time slot, the network topology is relatively static. The speed of ground users is distributed randomly to simulate the heterogeneous and diverse user characteristics in a real environment. For the *t*-th time slot, the current satellite, user, and content are denoted as $s^{t}$, $u^{t}$, and $o^{t}$, respectively.

### 2.3. Communication Model

We assumed that all edge users within the satellite coverage area could establish service with the satellite. We calculated the intersection point of the line connecting each satellite to the center of the Earth with the Earth’s surface, and then we computed the distance between the intersection point and the user as *d*. Each user selects the satellite with the minimum *d* to establish a communication link. It is easy to understand that, when the satellite is directly above the user, its projection coincides with the user, resulting in the highest communication quality. To represent the access relationship between users and satellites, we use the variable $\alpha ≜{\left[\alpha_{m,k}\right]}_{\forall (m,k)\in (M\times K)}$ as

(3) $$\alpha_{m,k}=\left\{\begin{array}{cc} 1, & {\mathrm{SUE}}_{k}\;\mathrm{is}\;\mathrm{served}\;\mathrm{by}\;{\mathrm{LEO}}_{m},\\ 0, & \;\mathrm{otherwise}.\; \end{array}\right.$$

Based on the real application scenario, we assumed that each edge user can only access one satellite. Therefore, the following constraints can be derived:

(4) $$\;(C1):\;\sum_{\forall m\in M}\alpha_{m,k}\leq 1,\forall k\in K.$$

Once ${\mathrm{SUE}}_{k}$ is served via ${\mathrm{LEO}}_{m},letW_{k}^{\mathrm{SUE}\;}$, which is the bandwidth allocated to ${\mathrm{SUE}}_{k}$, $p_{m,k}$ indicates the transmission power of this user. The orthogonal bandwidth assignment is assumed in this work based on which the signal-to-noise ratio (SNR) of ${\mathrm{SUE}}_{k}$ can be written as

(5) $$\gamma_{m,k}^{\mathrm{SUE}}=\frac{p_{m,k}h_{m,k}}{\sigma_{m}W_{k}^{\mathrm{SUE}\;}},$$

where $\sigma_{m}$ is the noise power density per Hz at ${\mathrm{LEO}}_{m}$. The achievable rate of ${\mathrm{SUE}}_{k}$ at ${\mathrm{LEO}}_{m}$ can be expressed as

(6) $$R_{m,k}^{\mathrm{SUE}}=W_{k}^{\mathrm{SUE}}\log_{2}\left(1+\gamma_{m,k}^{\mathrm{SUE}}\right)=W_{k}^{\mathrm{SUE}}\log_{2}\left(1+\frac{p_{m,k}h_{m,k}}{\sigma_{m}W_{k}^{\mathrm{SUE}}}\right).$$

Taking into account the LEO association decision, the achievable transmission rate of ${\mathrm{SUE}}_{k}$ can be described as

(7) $$R_{k}^{\mathrm{SUE}}\left(p,W^{\mathrm{SUE}},\alpha \right)=\sum_{\forall m\in M}\alpha_{m,k}R_{m,k}^{\mathrm{SUE}},$$

where $p≜{\left[p_{m,k}\right]}_{\forall m,k}$ and $W^{\mathrm{SUE}}≜{\left[W_{k}^{\mathrm{SUE}}\right]}_{\forall k}$. Regarding the communication rate demand at each SUE, the following constraint is introduced,

(8) $$\left(C2\right):\;R_{k}^{\mathrm{SUE}\;}\left(p,W^{\mathrm{SUE}\;},\alpha \right)\geq {\bar{R}}_{k}^{\mathrm{SUE}\;},\;\forall k\in K,$$

in which ${\bar{R}}_{k}^{\mathrm{SUE}}$ indicates the required transmission rate of ${\mathrm{SUE}}_{k}$.

### 2.4. Caching Model

Based on the characteristics of LEO satellite orbits, we assume that all satellites within the environment move in the same direction. Satellite access to edge users follows a Poisson distribution with an average arrival rate of λ. When an edge user accesses a satellite node, a content request is generated. Considering the segmented nature of content requests, we assume that, within one service cycle of a satellite, user requests can be completed. Therefore, in a single segment request, the edge user and fog satellite node are unique. The speed of satellites is dependent on the orbit altitude, and satellites at the same altitude move at the same speed.

We denote satellite nodes and edge users, respectively, as s={1,2,…,N} and u={1,2,…,K}. The requested content is represented as o={1,2,…,M}. We assume that the cache priority between different contents is equal, primarily considering user demands for cache decisions. We consider that each user may have preferences for several content types, with preference levels defined as $\alpha \in \left[0,1\right]$. A higher value of α indicates a higher preference level for the user, also implying a higher probability of the user requesting the corresponding content.

**Figure 2:**
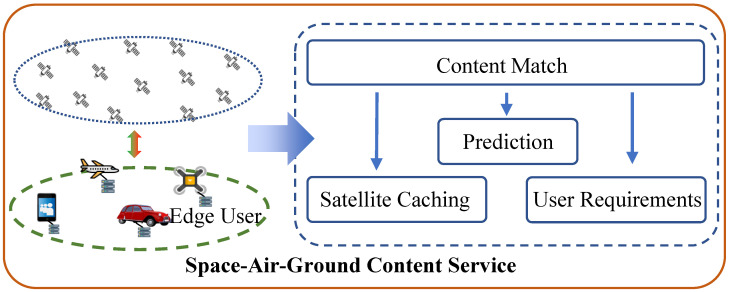
Cache structure.

The inter-satellite links of LEO satellites utilize laser for communication, offering an extremely high bandwidth and ultra-low latency, which can be negligible in the overall delay. Therefore, as shown in Figure 2 in our model, we assume that, when cached content exists in neighboring satellites, it also indicates a cache hit. If cached content is absent in all the satellites accessed by the current user and neighboring satellites, the satellite needs to request content from the cloud. In this case, due to the additional satellite-to-ground transmission process, we consider the transmission delay for cached content to double.

## 3. Implementation

### 3.1. Introduction to Deep Reinforcement Learning

Traditional reinforcement learning (RL) is a process in which the “agent” entity continuously, through trial and error, evaluates rewards and improves solutions in the unknown environment. Some classic methods include Q-learning, SARSA, etc. During the learning process, the “agent” selects corresponding actions based on the current state, obtains corresponding rewards, and transitions to the next state. It is worth noting that each action may affect the subsequent state. Through continuous iteration, the RL method aims to determine the optimal action in each state to obtain the largest reward in the target problem. Generally, reinforcement learning can be modeled as a Markov model: $MDP=\langle S,A,P,R,\gamma \rangle$, where *S* represents the set of states, *A* represents the set of actions, *P* represents the probability of selecting different actions in each state, and *R* represents rewards. The parameter γ is a discount factor used to discount future rewards into current rewards.

**Figure 3:**
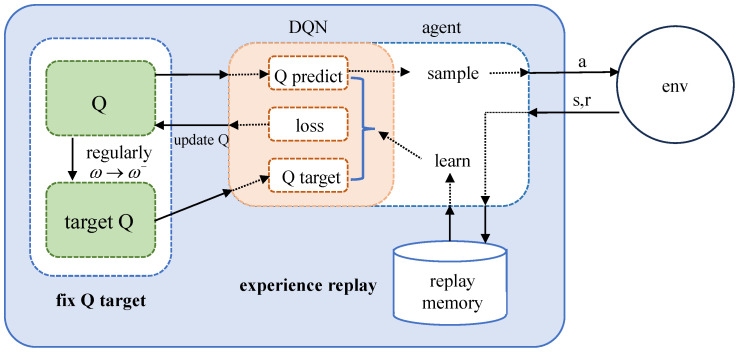
Process of DQN algorithm.

However, traditional RL methods can only handle limited and discontinuous states, which have certain limitations. With the combination of deep learning, the agent can learn to observe any state feature in the environment and perform corresponding decision training. The DQN algorithm is one of the classic DRL methods that has been improved on the basis of the Q-learning algorithm, and its process is shown in Figure 3.

As shown in Figure 3, the DQN algorithm adopts an experience replay mechanism. For any state, *s*, after taking an action, *a*, it will enter another state, $s^{\prime }$, and receive the corresponding reward, *r*. The quadruple $\langle s,a,r,s^{\prime }\rangle$ will form a sample and be stored in the replay buffer. After reaching the specified number, a batch of samples will be randomly selected for training. The DQN algorithm uses two Q-networks; one is the evaluation network, also known as the original network, which is responsible for controlling the “agent” and collecting experience, with weight parameters of ω, and the other is the target network, with weight parameters of $\omega^{-}$, which is used to calculate the time difference (TD) target, as shown in the following equation.

(9) $$y=r+\gamma \cdot \arg\max_{a}Q\left(s^{\prime },a;\omega^{-}\right)$$

The DQN algorithm uses the gradient descent method to update the weight parameters; the update formula is as follows:

(10) $$\omega_{t+1}=\omega_{t}+\alpha \left[r+\gamma \cdot \arg\max_{a}Q\left(s^{\prime },a,\omega^{-}\right)-Q\left(s,a,\omega^{-}\right)\right]\nabla Q\left(s,a,\omega^{-}\right)$$

During the update process, we can adapt the approach by fixing the weight parameters of the original network, updating the weight parameters of the target network, and regularly copying the target network weight parameters to the original network in order to enhance the fitting stability. However, the DQN algorithm involves the weight parameters of the target network in its own update process, which leads to the accumulation of positive errors and the overestimation of the Q value. In this regard, the Double DQN algorithm has been improved on the basis of the DQN algorithm by using the origin Q-network to obtain the best action, which, to some extent, solves the problem of overestimation. In the Double DQN algorithm, the calculation formula for the TD target is as follows:

(11) $$y=r+\gamma \cdot \arg\max_{a}Q\left(s^{\prime },a;\omega \right)$$

where *s* is the size of the transmitted content, and $R_{s,u},R_{s,s^{\prime }},R_{c,u}$ are the transmission rates between the fog satellite and users, the fog satellite and its adjacent satellite, and cloud service centers and users, respectively. We set different reward functions based on the transmission delay of cached content in different situations, which are shown in the following equation:

(12) $$r\left(t\right)=\left\{\begin{array}{cc} e^{-\lambda_{1}d_{1}} & f\in s(t)\\ e^{-(\lambda_{1}d_{1}+\lambda_{2}d_{2})} & f\in s_{n}\left(t\right)\\ e^{-\lambda_{3}d_{3}}\; & f\notin s\left(t\right)\mathrm{and}\;f\notin s_{n}\left(t\right) \end{array}\right.$$

where $\lambda_{1}+\lambda_{2}+\lambda_{3}=1,\lambda_{1}<\lambda_{2}\ll \lambda_{3}$. Therefore, the overall reward function is accumulated from the rewards of all the requested content on each satellite.

(13) $$R\left(t\right)=\sum_{Sat}\sum_{f}r\left(t\right)$$

### 3.2. Caching Hit Method

**Figure 4:**
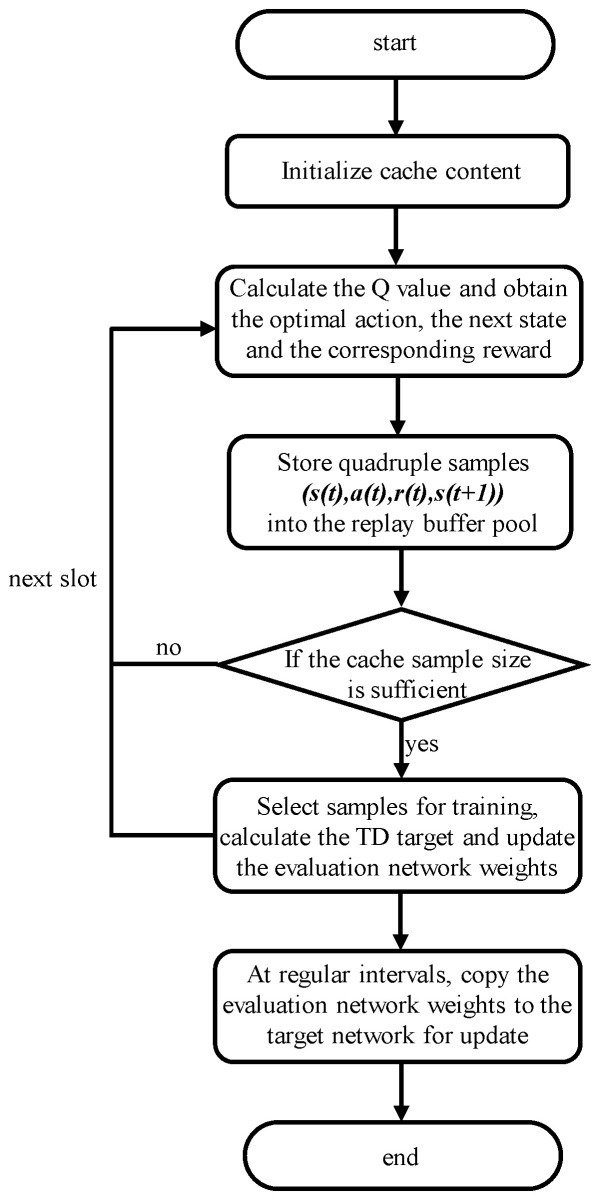
Flowcharts.

Based on the above settings, we propose a caching strategy based on Double DQN. A flowchart of the method is shown in Figure 4, where a detailed introduction to the algorithm is provided as follows:

1. Initialization: The local fog satellite selects *k* contents with a high request frequency as the cache content, while an adjacent satellite caches other *k* contents outside of the local fog satellite’s cached content.
2. In each time slot, observe the current state, s(t), calculate the Q value, and obtain the action, a(t), taken in that state. After executing the action, observe the new state, s(t+1), and obtain the reward, r(t).
3. Store the tuple (s(t),a(t),r(t),s(t+1)) as a sample in the replay buffer.
4. After samples of the buffer have reached a certain number, a random batch of them is selected for training. The TD target of all contents is calculated, and the weight parameters of the evaluation network will be updated with the gradient descent method.
5. After every certain number of steps, copy the weight parameters of the evaluation network to the target network.

### 3.3. Performance Benchmark

Our proposed approach (Section 3.2), referred to as the proposed method hereafter, was compared to three representative approaches, namely a Thompson sampling baseline, a Random baseline, and ε-greedy approaches to solving the caching problem. Thompson Sampling: In this method, the selection of content to cache on satellites is determined probabilistically using a Bayesian model. Each piece of content is chosen to be cached on a satellite by sampling from the posterior distribution, which is updated based on the historical hit rate and other relevant data. Content items are cached sequentially, with each decision influenced by the feedback obtained from previous cache hit outcomes.

1. Random algorithm: In this approach, the selection of content to cache on satellites is performed randomly without considering past performance or user preferences. Each piece of content is randomly assigned to a satellite for caching without any probabilistic model guiding the decision-making process. Content items are cached in no particular order, with each allocation being independent of previous cache hit outcomes or historical data.
2. ε-greedy algorithm: In this approach, the selection of content to cache on satellites combines both the exploration and exploitation strategies. With a probability of ε, a random content item is chosen for caching, allowing for the exploration of new content and satellite combinations. With the remaining 1−ε of the time, the algorithm exploits the known information by selecting the content with the highest estimated cache hit rate for caching on a satellite. This method balances between exploring new content possibilities and exploiting the currently known best options.
3. CAFR [33]: A cooperative caching scheme for edge computing that leverages FL and DRL to predict popular content and optimize caching strategies, this approach aims to improve cache hit ratios and reduce content transmission delays. It is currently the most outstanding method in the field of edge caching.

All experiments were implemented in Python 3.9.2 and conducted on a Windows machine equipped with an Intel Core i9-12900K processor (16 CPUs, 3.2 GHz), 32 GB of RAM, and an RTX3070 graphics card.

## 4. Simulation

In the simulation, we constructed a satellite network consisting of 66 satellites distributed across 6 orbital planes. On the ground, we employed Monte Carlo to generate 200 users following a Poisson-point-process (PPP) distribution for simulation settings. As shown in Table 1, the other parameters in our experiments were set according to references [34].

**Figure 5:**
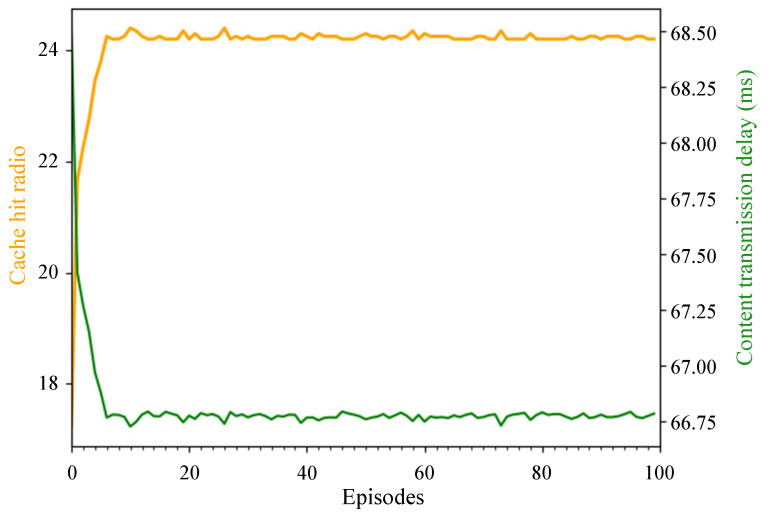
Cache hit radio and content transmission delay of each episode in training.

Firstly, we conducted training in the environment of Pytorch. Figure 5 illustrates one instance of the training process, showing that, as the training epochs increase, the decision outcomes of the DRL agent improve progressively. Around the tenth episode, the agent’s performance remains stable within a certain range of fluctuations. The latency stabilizes at around 66.75 ms, and the cache hit rate stabilizes at around 24%, demonstrating the stability and effectiveness of the algorithm.

**Figure 6:**
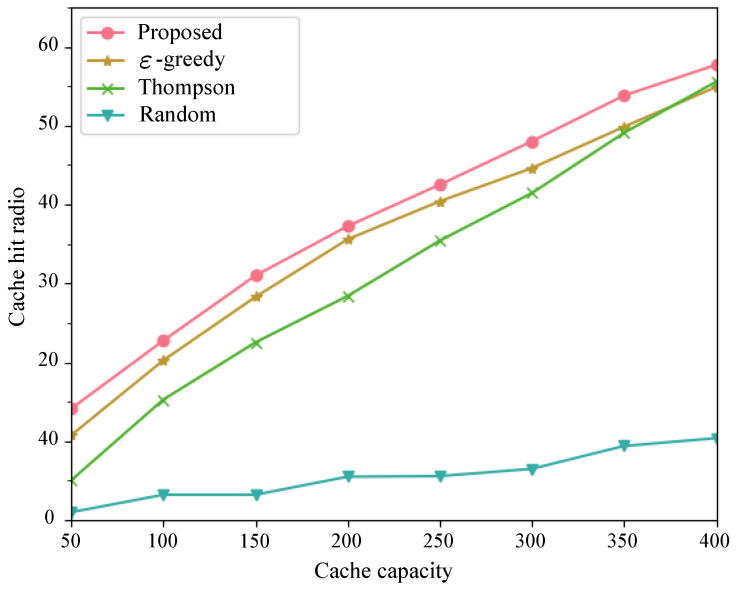
Cache hit radio with different cache capacities.

**Figure 7:**
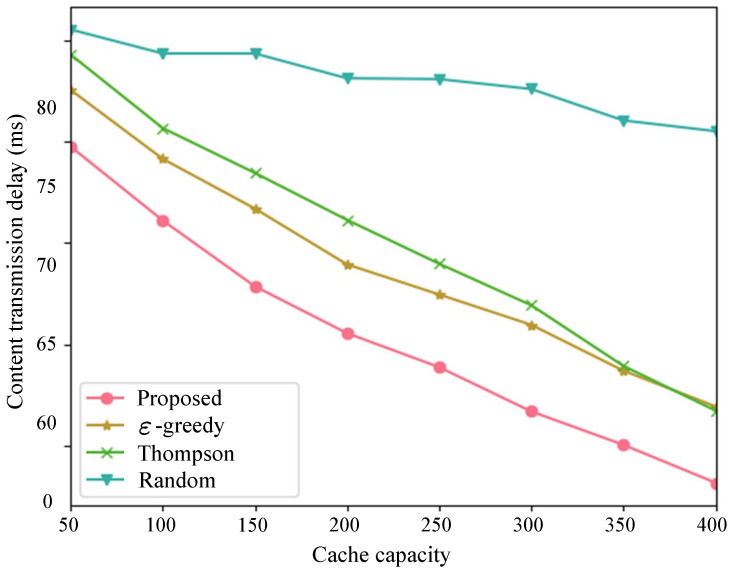
Content transmission delay with different cache capacities.

Subsequently, as depicted in Figure 6 and Figure 7, we compared the proposed method with the performance benchmark introduced in Section 3.3. We varied the cache capacity from 50 to 400 to obtain cache hit rates and content transmission delays under different cache capacities. With the increase in cache capacity, except for the random algorithm exhibiting the worst performance, the cache hit rates of the other three algorithms continuously increased, while the average content transmission delay continuously decreased. When the cache capacity was sufficiently large, the ε-greedy algorithm and Thompson algorithm showed similar performance, while our proposed method consistently demonstrated optimal performance.

**Figure 8:**
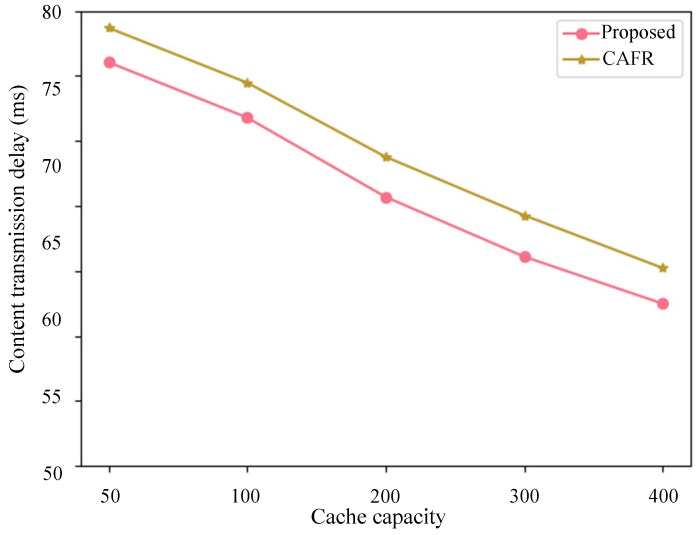
Content transmission delay compared to CAFR.

To demonstrate the distinctiveness and superiority of the proposed method over existing methods, we compared it with the most advanced method, CAFR, in the current edge-caching domain. The content-caching delay was primarily used as the evaluation metric. Figure 8 presents the comparative results, from which it can be inferred that the proposed method effectively addresses the satellite edge-caching issue.

## 5. Conclusions

This paper has addressed the issue of the content-caching strategy in the context of cloud–fog–edge collaboration scenarios in satellite internet services. We proposed a caching prediction scheme that considers the mobility characteristics of LEO satellites to enhance the cache hit rate of fog satellite nodes. Initially, we analyzed the characteristics of LEO constellations and edge users, establishing a network model. Subsequently, we introduced a caching prediction method based on Double DQN to improve the cache hit rate. The simulation results demonstrate that our proposed method can enhance the cache hit rate by 13% compared to baseline schemes. Based on the analysis, it can be concluded that, with the assistance of the Double DQN algorithm, the cache hit rate of edge-user cache requests can be effectively improved, thereby efficiently saving satellite networks’ bandwidth resources and reducing content-transmission latency.

## Figures and Tables

**Table 1:** Variable description.

Variable	Description
LEO satellite bandwidth	500 MHz
LEO satellite altitude	1000 km
LEO satellite antenna gain	40 dBi
UE antenna gain	30 dBi
Number of UEs	50–400
Number of Sats	66

## Data Availability

The data presented in this study are contained within the article.

## Abbreviations

The following abbreviations are used in this manuscript:

SFC	Satellite fog computing
Double DQN	Double Deep Q-Network
LEO	low Earth orbit
IoT	Internet of Things
VNI	Visual Networking Index
NDN	Named data networking
QoE	Quality of experience
AP	Access point
RL	Reinforcement learning
TD	Time difference
